# Population health, not individual health, drives support for populist parties

**DOI:** 10.1093/pnasnexus/pgac057

**Published:** 2022-05-19

**Authors:** Joost Oude Groeniger, Alexi Gugushvili, Willem de Koster, Jeroen van der Waal

**Affiliations:** Department of Public Health, Erasmus University Medical Centre, PO Box 2040, 3000 CA, Rotterdam, The Netherlands; Department of Public Administration and Sociology, Erasmus University Rotterdam, PO Box 1738, 3000 DR, Rotterdam, The Netherlands; Department of Sociology and Human Geography, University of Oslo, Postboks 1096, Blindern, 0317 Oslo, Norway; Nuffield College, University of Oxford, New Rd, Oxford OX1 1NF, UK; Department of Public Administration and Sociology, Erasmus University Rotterdam, PO Box 1738, 3000 DR, Rotterdam, The Netherlands; Department of Public Administration and Sociology, Erasmus University Rotterdam, PO Box 1738, 3000 DR, Rotterdam, The Netherlands

**Keywords:** health, support for populist parties, fixed effects models

## Abstract

Recent electoral shifts toward populist parties may have been partly driven by deteriorating health, although empirical evidence on this link is primarily confined to ecological designs. We performed both ecological- and individual-level analyses to investigate whether changes in health are associated with changes in the support for populist parties. Data were used on the strategic Dutch case, the only liberal democracy featuring leftist and rightist populist politicians in parliament for over a decade. We used: (a) fixed effects models to examine whether changes in the standardized mortality ratios and self-assessed health (SAH) in municipalities were associated with changes in the populist vote share in four parliamentary elections (2006/2010/2012/2017); and (b) 10 waves of panel data collected in 2008 to 2018 to investigate if changes in individual-level SAH were linked to movement in the sympathy, intention to vote, and actual voting for populist parties. The ecological analyses showed that: changes in municipality mortality ratios were positively linked to changes in the vote share of right-wing populist parties, while changes in the prevalence of less-than-good SAH were negatively associated with changes in the vote share for left-wing populist parties. The individual-level analyses identified no such associations. Our findings imply that support for populist parties may be driven by health concerns at the ecological, but not the individual, level. This suggests that sociotropic (e.g. perceiving population health issues as a social problem), but not egotropic (e.g. relating to personal health issues like experienced stigma), concerns may underlie rising support for populist parties.

Significance Statement Emerging scholarship implicates deteriorating health as an important predictor of support for populist parties, but empirical evidence on this link remains limited to ecological designs. This study is the first to examine whether changes in health affect support for populist parties at both an ecological and an individual level, and to do so for both left-wing and right-wing populist parties. The results show that changes in municipality mortality ratios are positively associated with changes in vote shares for right-wing populist parties, but not for their left-wing counterparts. Meanwhile, changes in the health of individuals are not associated with movement in the support for populist parties. This indicates that sociotropic, not egotropic, concerns may partially underlie support for right-wing populist parties.

## Introduction

One of the main developments in the global political landscape is undoubtedly the rise of populist political movements, parties, and campaigns. The best-known recent examples are the success of Donald Trump in the 2016 US presidential election and the vote for Brexit in the same year, although there have been other instances of populism becoming increasingly salient across the globe, including: the electoral success of far-right populist parties in northern (Sweden), western (France, the Netherlands), and eastern Europe (Poland and Hungary); the rise of far-left populist movements in southern Europe (Greece and Spain); religion-tinged populism in Asia (India); and antiestablishment campaigns in Latin America (Brazil) (1, 2). This surge in populism, especially its right-wing manifestations, has been met with great concern by public-health organizations (3) and leading medical journals (4), particularly since right-wing populist electoral outcomes may have profound implications for population health (5). Negative health consequences may include cuts to the budgets for: social- and health-care services (6); migrant care (5); and environmental policies (7). Most recently, a further example is the lack of an adequate response to the COVID-19 pandemic by right-wing populist politicians (8, 9). On the other end of the populist spectrum, left-wing parties can affect health by advocating for greater social expenditure, which is an important determinant of health (10, 11).

While the surge in populism can have profound implications for population health, emerging historical (for Italy and Germany) and contemporary (for the United States and the United Kingdom) analyses implicates deteriorating levels of population health as an important predictor of support for populist parties (12–17). Possible reasons for this association include communities blaming the political system for their poorer health (18), or the exploitation of this issue as a “political opportunity structure” by populist parties campaigning on health-related matters (19). Along with these supraindividual (ecological) associations, individual-level mechanisms might also be of importance. For example, citizens with lifestyle-related health problems (e.g. lung cancer and obesity), may feel looked down on and stigmatized (20, 21), fueling resentment that drives support for populist parties. In addition, citizens with poor health are more likely to have (possibly negative) experiences with the health care system or social security system (e.g. when applying for disability benefits) and may feel that the system has let them down. Lastly, citizens with poor health would also have more personal benefit from medical care expansions especially campaigned on by left-wing populist parties, such as SYRIZA in Greece and the Socialist Party (SP) in the Netherlands (22, 23), but also by some right-wing ones in countries on the European continent, such as the Dutch Party for Freedom (PVV) and Polish PiS (24). Consequently, understanding if and how health affects support for right- and left-wing populist parties and candidates requires both ecological- and individual-level analyses. Yet, so far the empirical evidence almost exclusively refers to the role of health in right-wing populism and is primarily confined to ecological designs.

The analyses of historical data from the beginning of the 20th century have shown an association between worsening mortality rates and the rise of Fascism in Italy and Nazism in Germany (16, 17). Contemporary studies based on ecological designs have also linked the fall in life expectancy (13), rising death rates (25), and the increase in health inequalities observed in some counties in the United States to voting for Donald Trump in the 2016 presidential election (12). Voting for Trump has been linked to the chronic use of prescription opioid drugs (14), poor and worsening psychological wellbeing (26), and various other health indicators (27). Evidence on the implications of deteriorating health for populism also exists in relation to the 2016 Brexit referendum, with increases in so-called “deaths of despair” between 2007 and 2016 and in suicides from 2015 to 2017 linked to “leave” votes (15, 28). The main shortcoming of such ecological studies, however, is that they cannot be used to determine whether individuals abandon their previous mainstream political preferences for populist options due to their worsening health (29). To the best of our knowledge, only two studies have investigated the individual-level association between health and populist support. These used data from a large number of European countries and showed that individuals with disabilities and those with poorer self-assessed health (SAH) were more likely to vote for right-wing populist parties than their counterparts with no disability and better SAH (30, 31). Nevertheless, the cross-sectional design of these studies limits the possibility to make causal inferences.

Our study advances the research described above in three interrelated ways. First, it estimates both ecological- and individual-level effects in a single country, because changes in health at both of these levels can affect support for populist politicians. While such an analysis would ideally also focus on how those levels interrelate, as to investigate whether changes in community health affect changes in individual-level voting patterns, this was not possible in the current study as data on neighborhoods or municipalities were not available in the individual-level dataset due to privacy regulations. Second, the individual-level effects are estimated using unique representative panel survey data, which answers a recent call to improve causal inference by using longitudinal data on both health status and political preferences (31). Third, our focus is on the link between changes in health and support for populism not only at the right but also at the left of the political spectrum. This is crucial, as left-wing populists are likely to advocate increasing the level of investment in solidarity-based health care systems (32). To the best of our knowledge, the Netherlands is the only liberal democracy where both leftist and rightist populist parties have not only been represented in parliament for over a decade, but have also garnered substantial support. Moreover, the largest and most dominant ones—the Socialist Party (SP) on the left and Party for Freedom (PVV) on the right—both campaign on abolishing health insurance deductibles and cancelling cuts in healthcare, especially regarding care for the elderly (33, 34).

Because trends in population health in the Netherlands have been much more favorable in the last decade compared to those in the United States and the United Kingdom, the countries where most of the existing evidence stems from, our empirical investigation can be considered as a strict test of the relation between changing health and changing populist support. Using both high-quality panel survey data (2008 to 2018) and municipal-level data covering four parliamentary elections (2006/2010/2012/2017), we aim to answer the question: *Do changes in health affect support for populist parties?*

## Methods

### Ecological data

Municipal-level data were retrieved from Statistics Netherlands (CBS), the National Institute for Public Health and the Environment (RIVM), and the Dutch Electoral Council (*Kiesraad*) (35, 36). Municipalities that did not change their administrative boundaries during four parliamentary elections (2006 November 22; 2010 June 9; 2012 September 12; and 2017 March 15) were included. This produced a sample of 327 municipalities.

The outcome variables were calculated as the share of the vote for right- and left-wing populist parties separately: votes for right-wing/left-wing populist parties divided by the total number of votes cast (excluding votes for populist parties at the other end of the political spectrum). Populist parties were defined using the standard populist party classification (37, 38). Right-wing populist parties consisted of: the “Party for Freedom” (PVV) and “List Pim Fortuyn” (LPF) in 2006; the PVV and “Proud of the Netherlands” (TON) in 2010; the PVV and “Democratic Political Turning Point” (DPK) in 2012; and the PVV, “Forum for Democracy” (FvD) and “For Netherlands” (VNL) in 2017. Only one left-wing populist party stood in those elections, the “Socialist Party” (SP).

The explanatory variables consisted of age- and sex-standardized mortality ratios (SMR), and an aggregated measure of the prevalence of less-than-good self-assessed health (prSAH) in residents age 19+. SMR were calculated as the ratio of the observed over the expected number of deaths, using the national-level mortality rates in 2000 as the standard population. We calculated a mean SMR for the 3 or 4 years preceding the election year to flatten year-to-year random fluctuations in the small municipalities in the sample. Mortality ratios in the election year were excluded (included) if elections were held during the first (last) 6 months of the year. This left us with four mean SMR measures for the following years: 2003 to 2006, 2007 to 2009, 2010 to 2012, and 2013 to 2016. prSAH was estimated using survey data and small-area estimation techniques, which are described in more detail elsewhere (39). These data were only available for the years 2012 and 2016.

Referring to previous ecological studies, additional time-varying covariates were included to adjust for sociodemographic characteristics (based on administrative data collected by Statistics Netherlands) that are likely to be related to both health and political preference: population density; percentage of one-person households; percentage of residents age 65+; median standardized income; average home value; percentage of people unemployed; percentage of less educated; and percentage of non-Western residents. Other covariates considered, but subsequently excluded due to multicollinearity, were: average household size; the percentage of households with children; and the percentage of married residents. All the covariates were lagged by 2 years compared to the years of the elections, save in relation to the median standardized income where, because of data availability issues, we used that from 2005, 2008, 2010, and 2014. We ran robustness checks with the covariates lagged by 1 year and by 3 years; the results were similar in both cases.

### Individual-level data

The individual-level analyses were conducted using data from the Longitudinal Internet Studies for the Social sciences (LISS) panel (administered by CentERdata at Tilburg University in the Netherlands). This panel is a true probability sample, which was produced by extracting a random sample of 10,150 private households from the Statistics Netherlands population register. The selected households were approached via letter, telephone call, and/or home visit. People were approached up to 15 times if necessary to minimize the number of nonresponses (40). A total of four refreshment samples have been added since the panel was established in 2007, some of which oversampled groups that are difficult to reach in order to improve representativeness. If required, CentERdata provides participants with an Internet connection and computer to enable them to complete the LISS questionnaires and makes a special effort to contact inactive panel members. Monetary compensation is awarded to the participants for their involvement. In 2019, the average individual response rate was 80.4%. Household data are updated monthly and the participants complete annual repeat core questionnaires to monitor changes in their lives. The LISS panel complies with all relevant ethical regulations (41).

We used the following LISS Core Studies in this research: “Politics & Values” (surveyed every December); “Health” (every November); and “Economic Situation” (every June). All of these data were obtained annually from 2008 to 2018 (except for 2014, when no data were collected). Additional background-characteristic data were also acquired every June. We only included observations from participants who had reached the age of 25, because this makes it more likely that they have finished their education and entered the workforce (limiting the possibility that changes in these political-preference determinants would affect the results of our analyses). If available, we included data on every individual aged 25 or older. This produced a sample of 9,234 unique individuals and 45,638 repeated observations.

We included three outcomes: sympathy for populist parties; intention to vote for populist parties (both measured annually); and actual (reported) voting for populist parties (only measured after real parliamentary elections). Sympathy was measured by asking the participants on a scale from 0 (very unsympathetic) to 10 (very sympathetic): “What do you think of [populist party]?.” Depending on the year, the right-wing populist parties standing included the PVV, TON, DPK, and FvD; the SP was the only left-wing populist party up for election in all the years considered. If multiple populist parties stood, we used the highest sympathy score provided (a higher score indicated more sympathy). Intention to vote was measured by asking: “If parliamentary elections were held today, which party would you vote for?” Actual voting for populist parties was measured by asking: “For which party did you vote in the parliamentary elections of [date/year]?” For both outcomes, the responses were coded as 1 if the participants chose one of the right-wing or left-wing populist parties, or as 0 if they opted for a different party or blank vote. If participants indicated that they did not vote, or if the participants opted for a populist party at the other end of the political spectrum than the outcome considered, the responses were coded as missing (e.g. in models considering right-wing populist voting, left-wing populist voters and nonvoters were excluded from the analysis).

In total, two explanatory variables were included: SAH and mental health (MHI-5). SAH was measured by asking: “How would you describe your health, generally speaking?.” The answer categories were poor, moderate, good, very good, or excellent (reverse coded, meaning a higher score indicates worse SAH). MHI-5 was measured using the validated five-item version of a questionnaire that asks how the respondents’ mental health has been over the previous 4 weeks (42). Each answer was measured on a six-point scale. A total mental health score was calculated as the mean of the scores obtained from the responses to the five-item questionnaire (reverse coded, with a higher score indicating worse mental health).

We included several time-varying covariates to adjust for potential confounding factors: age; partner (yes or no); number of children; net monthly household income (with income top-coded at €10,001 to limit the influence of extremes); paid work (yes or no); and financial difficulties (“Can you indicate on a scale from 0 to 10 how hard or how easy it is for you to live off your income”).

### Statistical analysis

For both the ecological and individual-level analyses, we estimated random effects models to investigate the association between health and support for populist parties, and fixed effects models to investigate the association between changes in health and changes in populist support. The latter models account for any time-invariant characteristics that differ across individuals (or municipalities), such as gender and childhood circumstances, reducing the likelihood of potential confounders in the estimated associations. Hausman specification tests demonstrated, for both the municipality and individual level, that fixed effects models were preferred over random effects models in the circumstances of this research. The results from the random effects models are therefore provided in Tables S1 and S2 (Supplementary Material), while those from the fixed effects models are set out below. The following fixed effects model was used:

$$\begin{equation*}
Po{p_{it}} = {\rm{ }}{\mu _t} + {\rm{ }}{\beta _1}healt{h_{it}} + {\rm{ }}{\beta _2}{x_{it}} + {\rm{ }}{\alpha _i} + {\rm{ }}{\epsilon _{it}},
\end{equation*}
$$

where *Pop_it_* indicates the outcome of interest for municipality/individual *i* at time *t; health_it_* represents the explanatory variable; *x_it_* is a vector of the additional time-varying covariates; and ϵ_it_ is the error term. *μ_t_* accounts for time effects that are fixed for all municipalities/individuals, while *α_i_* controls for time-invariant municipality/individual characteristics. At the individual level, we opted for linear probability models for the binary outcomes to aid interpretation (the results from the fixed effects logistic models were similar to those of the linear probability models and are reported in Table S3, Supplementary Material). All the models were adjusted for both the time fixed effects and the time-varying covariates listed above. Robust standard errors were used to account for clustering at the municipality/individual level.

Following the theoretical mechanisms described in the introduction, it is possible that changes in health are more strongly related to changes in support for populist parties among individuals/communities with a worse health status, because such mechanisms may convey a greater effect among those who may arguably feel more “deprived” of good health (43). Scrutinizing this nonlinearity between units in fixed effects models can be achieved by including a quadratic term in the fixed effects regression model (McIntosh and Schlenker (44) and Giesselmann and Schmidt-Catran (45) provide a more detailed explanation of why a quadratic term in fixed effects models captures nonlinearity between units rather than within units). Therefore, we adopted a two-step approach for all the analyses. First, we fitted the models that included only the linear term of the health variable. Second, we added a quadratic term of the health variable to the models to allow for a nonlinear relationship. Whenever we observed nonlinear effects, we subsequently estimated marginal effects to investigate this heterogeneity across levels of health (the health variables were grand-mean centered to aid interpretation).

## Results

### Ecological analysis

Municipal characteristics during the first and fourth elections covered in this study are set out in Table 1. The vote share of right-wing populist parties almost tripled between 2006 and 2017 (7.0% in 2006; 17.2% in 2017), while that for such parties on the left fell by a third (16.5% in 2006; 11.4% in 2017).

**Table 1.** Baseline characteristics of the municipalities and LISS participants.

	First wave		Last wave	
	Mean (SD)	Min–Max	Mean (SD)	Min–Max
Ecological analysis				
SMR	87.9 (9.7)	58–118	71.5 (7.9)	40–98
PrSAH	23.5 (3.2)	17–38	24.2 (3.3)	17–37
Votes for right-wing populist party	7.0 (3.1)	2–20	17.2 (5.3)	7–45
Votes for left-wing populist party	16.5 (5.0)	1–35	11.4 (5.1)	1–32
Population density	829.1 (994.2)	25–5,674	863.0 (1051.1)	25–6,289
Percentage of one-person households	27.8 (7.0)	18–58	31.5 (6.6)	19–62
Percentage of residents age 65+	41.2 (4.4)	22–55	49.2 (5.0)	28–61
Median standardized income	18.5 (1.6)	14–24	22.2 (1.9)	17–33
Average home value	146.0 (38.6)	77–363	219.9 (54.2)	119–534
Percentage unemployed	5.0 (1.1)	4–9	6.1 (1.1)	4–12
Percentage low educated	0.4 (0.1)	0–1	0.3 (0.1)	0–0
Percentage non-Western residents	5.2 (5.0)	1–35	6.3 (5.8)	1–37
Individual analysis				
SAH (excellent—poor)	2.8 (0.8)	1–5	2.9 (0.8)	1–5
Mental health (excellent—poor)	2.3 (0.8)	1–6	2.2 (0.8)	1–6
Sympathy for right-wing populist party	3.4 (2.6)	0–10	3.4 (2.8)	0–10
Sympathy for left-wing populist party	5.3 (2.0)	0–10	4.8 (2.4)	0–10
Intention to vote for right-wing populist party	14.2%		19.5%	
Intention to vote for left-wing populist party	13.6%		10.9%	
Voted for right-wing populist party	13.4%		11.6%	
Voted for left-wing populist party	11.6%		10.6%	
Age	50.5 (13.6)	25–94	55.9 (15.6)	25–100
Partner (yes)	79.6%		69.2%	
Number of children	0.8 (1.1)	0–7	0.6 (1.0)	0–6
Household income	1530.9 (1083.9)	0–10,001	1763.9 (1054.5)	0–10,001
Work (yes)	61.0%		50.1%	
Financial difficulties	6.5 (2.0)	0–10	7.0 (1.9)	0–10

**Fig. 1.**
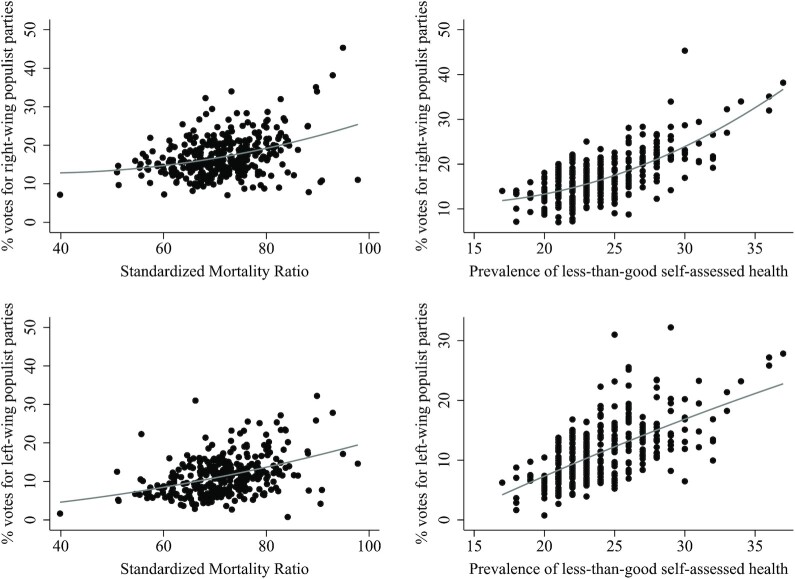
Association between municipality health and the vote shares of right-wing (upper panels) and left-wing (lower panels) populist parties in the 2017 House of Representatives election in the Netherlands (including quadratic regression line).

Figure 1 plots the unadjusted association between municipality health and populist voting in the 2017 parliamentary election. In line with previous studies from the United States and the United Kingdom, this shows that higher mortality ratios are linked to a higher vote share for both right-wing and left-wing populist parties. A higher prSAH in the municipality is also related to a higher vote share for populist parties. Furthermore, Figure 1 demonstrates that the positive association between municipality health and the populist vote share (especially the right-wing populist vote share) is stronger in municipalities with higher mortality ratios.

**Fig. 2.**
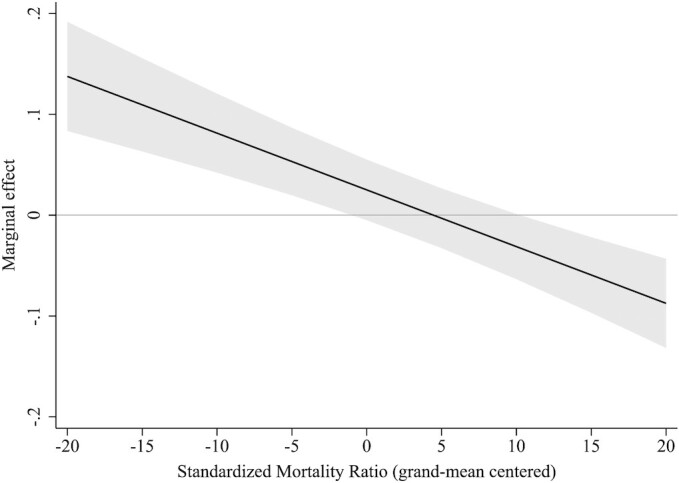
Marginal effect (including 95% CIs) of changes in mortality ratios on changes in vote shares for right-wing populist parties.

**Fig. 3.**
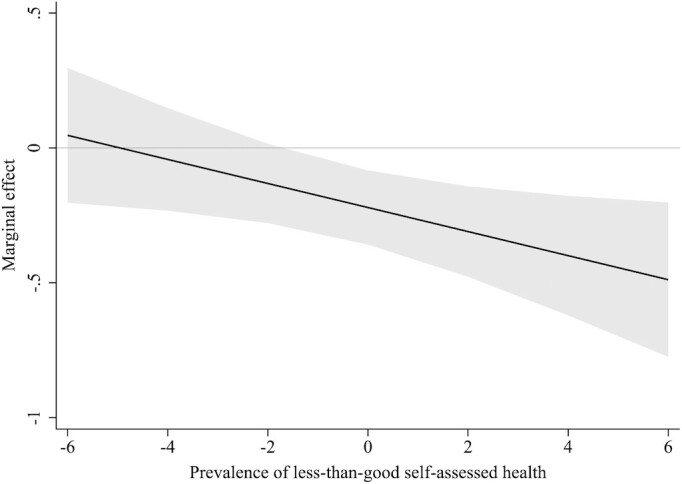
Marginal effect (including 95% CIs) of changes in prSAH on changes in vote shares for left-wing populist parties.

Results from the random effects models also showed a clear association between levels of municipality health and vote shares for right- and left-wing populist parties across the four parliamentary elections included (Table S1, Supplementary Material). However, Hausman specification tests demonstrated that fixed effects models were preferred over random effects models. Results from fixed effects models are set out in Table 2 and indicate that a change in SMR is not linearly associated with a change in right-wing or left-wing populist voting, while an increase in the prSAH is linearly associated with a decrease in left-wing populist voting, but not associated with a change in right-wing populist voting (Model 1). The introduction of quadratic terms indicates that there is a nonlinear association between a change in SMR and a change in right-wing populist voting, but not for left-wing populist voting; meanwhile, for prSAH there is a nonlinear association with left-wing populist voting, but not with right-wing populist voting (Model 2). Figure 2 visualizes the marginal effects of the nonlinear association between a change in SMR and a change in right-wing populist voting. It shows that a rise in SMR is associated with an increase in voting for right-wing populist politicians in municipalities with below-average mortality ratios, while it is associated with a decrease in voting for right-wing populist politicians in municipalities with above-average mortality ratios. Figure 3 visualizes the marginal effects of the nonlinear association between a change in prSAH and a change in left-wing populist voting. It shows that a higher prSAH is associated with a decrease in voting for left-wing populist politicians in municipalities with average or above-average levels of less-than-good SAH. Because data on prSAH were only available for the elections of 2012 and 2017, we additionally analyzed the association between changes in SMR and changes in populist voting restricted to these two elections, which showed comparable results as those covering all four elections (Figure S1, Supplementary Material).

**Table 2.** Ecological associations between changes in municipality health and changes in the vote shares of populist parties.

	Model 1				Model 2			
	b	95% CI		*P*-value	b	95% CI		*P*-value
Votes for right-wing populist party								
Mortality ratios^a^	0.009	−0.014	0.032	0.457	0.025	−0.005	0.055	0.102
Mortality ratios-squared^a^					−0.003	−0.004	−0.002	0.000
PrSAH^b^	0.020	−0.117	0.156	0.778	0.020	−0.113	0.152	0.772
PrSAH^b^					0.000	−0.020	0.020	0.999
Votes for left-wing populist party								
Mortality ratios^a^	0.005	−0.020	0.030	0.703	0.005	−0.019	0.030	0.680
Mortality ratios-squared^a^					0.000	−0.001	0.001	0.920
PrSAH^b^	−0.236	−0.382	−0.091	0.002	−0.221	−0.359	−0.083	0.002
PrSAH^b^					−0.022	−0.042	−0.003	0.024

Note: All the models were adjusted for year fixed effects and the following covariates: population density, percentage of one-person households, percentage of residents aged 65+, median standardized income, average home value, percentage of unemployed, percentage of low educated, and percentage of non-Western residents.

a The analysis included the elections in 2006, 2010, 2012, and 2017.

b The analysis included the elections in 2012 and 2017.

### Individual-level analysis

At the individual level, and like the results from the ecological analyses, the random effects models showed an association between poorer health and more support for both right-wing and left-wing populist parties between 2008 and 2018 (Table S2, Supplementary Material). The results from the fixed effects models were not, however, in line with those of the ecological analyses: changes in SAH and mental health were not (linearly or nonlinearly) linked to changes in sympathy, intention to vote, or actually voting for populist parties (Table 3).

**Table 3.** Individual associations between changes in health and changes in the support for populist parties.

	Model 1				Model 2			
	b	95% CI		*P*-value	b	95% CI		*P*-value
Sympathy for right-wing populist party								
SAH	0.013	−0.032	0.058	0.568	0.014	−0.031	0.060	0.533
SAH-squared					0.008	−0.023	0.038	0.629
Mental health	−0.009	−0.046	0.028	0.641	−0.010	−0.055	0.036	0.683
Mental health-squared					0.001	−0.024	0.026	0.957
Sympathy for left-wing populist party								
SAH	−0.011	−0.052	0.030	0.602	−0.015	−0.056	0.026	0.479
SAH-squared					−0.021	−0.048	0.007	0.143
Mental health	0.020	−0.015	0.054	0.268	0.014	−0.027	0.054	0.504
Mental health-squared					0.006	−0.018	0.030	0.626
Intention to vote for right-wing populist party								
SAH	−0.002	−0.009	0.006	0.702	0.000	−0.009	0.008	0.956
SAH-squared					0.004	−0.001	0.010	0.113
Mental health	0.003	−0.004	0.010	0.449	0.000	−0.008	0.008	0.978
Mental health-squared					0.003	−0.002	0.008	0.289
Intention to vote left-wing populist party								
SAH	0.004	−0.004	0.012	0.292	0.004	−0.004	0.013	0.335
SAH-squared					0.000	−0.006	0.005	0.922
Mental health	−0.001	−0.010	0.007	0.760	−0.004	−0.012	0.005	0.437
Mental health-squared					0.002	−0.004	0.008	0.432
Voted for right-wing populist party^a^								
SAH	0.004	−0.010	0.018	0.560	0.006	−0.009	0.021	0.449
SAH-squared					0.006	−0.003	0.016	0.177
Mental health	−0.002	−0.016	0.013	0.809	0.001	−0.015	0.018	0.865
Mental health-squared					−0.003	−0.013	0.006	0.510
Voted for left-wing populist party^a^								
SAH	0.006	−0.010	0.023	0.465	0.007	−0.010	0.024	0.432
SAH-squared					0.003	−0.008	0.014	0.617
Mental health	0.009	−0.006	0.024	0.255	0.007	−0.011	0.025	0.426
Mental health-squared					0.002	−0.009	0.013	0.759

Note: a higher score on the health variables indicates poorer health. All the models were adjusted for year dummies and the following covariates: age, partner, number of children, log household income, work, and financial difficulties.

a The analysis only included data corresponding to the elections in 2010, 2012, and 2017.

## Discussion

The changing political landscape has stimulated emerging scholarship on the role of health in the rise of populism, especially its right-wing manifestation, in different parts of the world. To the best of our knowledge, this study is the first to examine whether changes in health affect support for populist parties on the left and right of the political spectrum and at both an ecological and an individual level. At the ecological level, we found that changes in mortality ratios were positively associated with changes in the vote share for right-wing populist parties in municipalities with below-average mortality ratios, while changes in the prSAH were negatively associated with changes in the vote share for left-wing populist parties at municipalities with an above-average prSAH. At an individual level, changes in self-assessed physical and mental health were not linked to either changes in sympathy for populist parties, or to the intention to vote for, or actually voting for them.

Complementing ecological analyses with those at the individual level is essential for uncovering why changes in health are associated with increased populist support, as both ecological and individual-level theories may explain this relationship. The lack of any individual-level associations between self-reported physical and mental health and support for populist parties indicates that people do not choose to vote for populist politicians because of changes in their own health status. This contradicts the hypothesis that populist voting is a potential effect of the resentment toward mainstream political parties, which increasingly frame health and wellbeing as a matter of individual responsibility (20, 21). While we did observe associations between SAH and support for populist parties in random effects models, these were absent in fixed effects models. This suggests that the observed cross-sectional link between individual health and populist support reported in two recent studies (30, 31) is likely spurious. Indeed, other factors correlated with both health and support for populist politicians (e.g. childhood circumstances and socialization, personality traits, and multidimensional socioeconomic position) are more probable drivers of the observation that populist voters have poorer health than their nonpopulist voting counterparts (46–49). These findings would be strengthened if they were corroborated when using objective measures of health (as is done in the ecological analyses using mortality ratios). While self-reported health taps into the perception of, rather than actual, health, and it is this perception that is arguably behind the support for populist parties, those with a particularly high risk of poor health (e.g. those who suffer from substance abuse) are least likely to participate in survey research. This may have resulted in an underestimation of the effects, albeit only if the same people do vote in elections.

Several ecological mechanisms may explain why changes in population health are linked to the vote share of populist parties, which our study is partially able to tease out because of its inclusion of parties at both the right and left of the political spectrum. A total of two mechanisms are often invoked by extant studies: (a) communities may blame the political “status-quo” for health problems and switch their political allegiance toward populist political parties and leaders; and (b) populist parties may benefit from population health issues by campaigning on them in deprived communities (19, 20). While the former mechanism would have predicted a higher vote share for both right- and left-wing populist parties in areas with more health problems, the latter would have anticipated greater left-wing voting, particularly because campaigning on health has most markedly been a feature of left-wing populists in the Netherlands. The absence of a clear positive association with left-wing populist voting—or in the case of SAH even the presence of a negative association—suggests that the higher health budgets proposed in the parties’ manifestos are unlikely to account for the ecological-level relationship between deteriorating health and populist support.

Contrary to our expectations, we observed that increasing municipal mortality ratios were only related to an increase in right-wing populist voting in municipalities with below-average mortality ratios. In other words, our results suggest that deteriorating population health is only related to a higher vote share for right-wing populist parties among communities that have relatively good population health, although these results were not observed for the prevalence of SAH. Since previous studies have not investigated the nonlinearity of the relationship between deteriorating population health and populist support, we are unable to verify whether this finding is typical to the Dutch context or whether it can also be observed in other countries. Speculating about potential explanations for these results, it may be that the higher mortality rates become more visible and are particularly felt in communities characterized by relatively high levels of health. This corroborates evidence indicating that the appeal of right-wing populist parties might be present even in prosperous environments (50), and that the threat to existing status and not actual hardship might explain support for Donald Trump in the 2016 US presidential election (51).

Although we conducted both ecological and individual-level analyses, our data did not enable us to combine them and identify any cross-level (interaction) effects. Future research should, therefore, consider whether deteriorating population health (i.e. on a collective level) is linked to increased support for populist parties on an individual level, or whether it moderates individual-level associations. This would allow researchers to further unravel if and why failing health is related to increased populist support, and make inferences about the relationship between community health and individual-level populist support without the risk of ecological fallacy (52). While our findings suggest that recent electoral shifts toward populist parties may have, to some extent, come about due to sociotropic anxieties (i.e. perceiving unfavorable mortality ratios to be a social problem), but not because of egotropic concerns (i.e. personal health problems), this will be strengthened if it can be corroborated by studies that relate both types of concern to individual-level populist support. Furthermore, given that the individual-level data cover a random sample of the Netherlands, large municipalities are likely strongly represented in these data (37% of the Dutch population resides in the 32 largest municipalities). Contrarily, in the ecological analysis all municipalities are included and weighted equally, which makes it difficult to directly compare the ecological and individual-level results.

Overall, our analyses on the strategically selected Dutch case found some evidence for a relationship between changing health and support for populist parties at an ecological level, but not at the individual level. Future research could establish this more firmly using even stricter methodological designs, and could also scrutinize how far our findings travel beyond the Dutch case.

## Supplementary Material

pgac057_Supplemental_File

## Data Availability

The ecological data used in this study are freely available online. The individual level data used in this study are stored and made available for reuse by CentERdata in their data repository (https://www.dataarchive.lissdata.nl), which has received the international CoreTrustSeal. Data from this database are available for academic research purposes under the conditions that users must sign a statement on statements.centerdata.nl, certifying that information on individuals and households will not be passed on to others and that all data will be treated as confidential. All analyses were conducted in Stata/MP 16.1 (StataCorp, College Station, TX). The code used to generate the results can be obtained from the corresponding author.
