# Supplementary material for: Population health, not individual health, drives support for populist parties
Source: PNAS Nexus. 2022 May 19;1(3):pgac057. doi: 10.1093/pnasnexus/pgac057 (PMC9896918; doi:10.1093/pnasnexus/pgac057)
Supplement: pgac057_Supplemental_File [file pgac057_supplemental_file.pdf]

Supplementary Information "Population health, not individual health, drives support for populist parties"

Table S1. Longitudinal associations between municipality health and the vote shares of populist parties estimated with random effects models.

	**Model 1**				**Model 2**			
**Outcome and health variables**	**b**	**95% CI**		***p*-value**	**b**	**95% CI**		***p*-value**
Votes for right-wing populist party								
Mortality ratios [a]	0.047	0.022	0.073	<0.001	0.062	0.038	0.087	<0.001
Mortality ratios-squared [a]					-0.003	-0.004	-0.002	<0.001
Prevalence of less-than-good self-assessed health [b]	0.647	0.551	0.742	0.000	0.582	0.484	0.680	0.000
Prevalence of less-than-good self-assessed health [b]					0.040	0.024	0.055	0.000
Votes for left-wing populist party								
Mortality ratios [a]	0.047	0.024	0.071	<0.001	0.046	0.023	0.070	<0.001
Mortality ratios-squared [a]					0.000	0.000	0.001	0.525
Prevalence of less-than-good self-assessed health [b]	0.482	0.376	0.587	0.000	0.462	0.351	0.572	0.000
Prevalence of less-than-good self-assessed health [b]					0.010	-0.007	0.027	0.252

Note: A higher score on the health variables indicates poorer health at the municipality level.
[a] The analysis included the elections in 2006, 2010, 2012 and 2017.
[b] The analysis included the elections in 2012 and 2017.

Figure S1. Marginal effect (including 95% CIs) of changes in mortality ratios on changes in vote shares for right-wing populist parties only including the elections in 2012 and 2017.

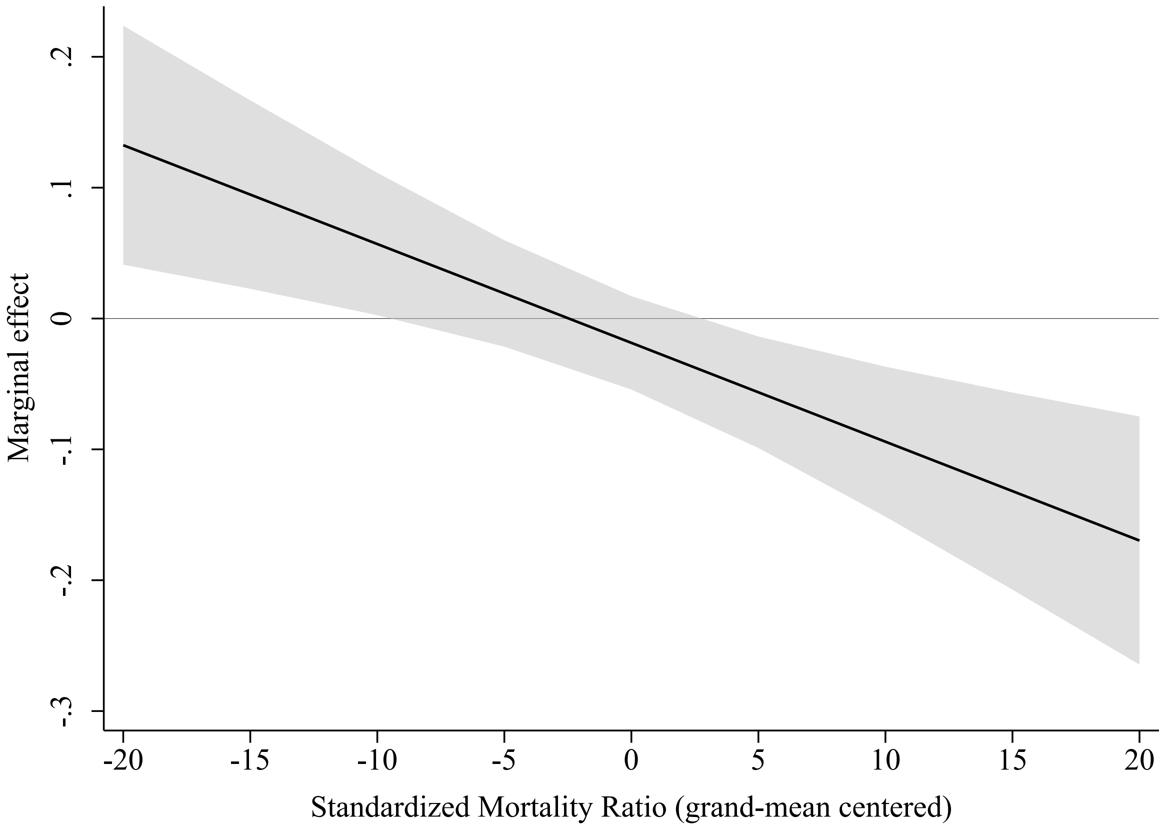

Table S2. Longitudinal associations between individual health and the support for populist parties estimated with random effects models.

	Model 1				Model 2			
**Outcome and health variables**	**b**	**95% CI**		***p*-value**	**b**	**95% CI**		***p*-value**
Sympathy for right-wing populist party								
Self-assessed health	0.028	-0.003	0.059	0.078	0.029	-0.002	0.061	0.069
Self-assessed health-squared					0.006	-0.016	0.027	0.615
Mental health	0.003	-0.025	0.030	0.842	0.001	-0.033	0.035	0.963
Mental health-squared					0.002	-0.018	0.022	0.840
Sympathy for left-wing populist party								
Self-assessed health	0.012	-0.016	0.040	0.402	0.011	-0.017	0.040	0.427
Self-assessed health-squared					-0.002	-0.022	0.018	0.841
Mental health	0.051	0.026	0.076	<0.001	0.066	0.035	0.096	<0.001
Mental health-squared					-0.015	-0.033	0.003	0.101
Intention to vote for right-wing populist party								
Self-assessed health	0.012	0.006	0.018	<0.001	0.014	0.008	0.020	<0.001
Self-assessed health-squared					0.008	0.003	0.012	<0.001
Mental health	0.007	0.002	0.012	0.006	0.002	-0.005	0.008	0.583
Mental health-squared					0.006	0.002	0.010	0.002
Intention to vote left-wing populist party								
Self-assessed health	0.022	0.016	0.028	<0.001	0.023	0.017	0.029	<0.001
Self-assessed health-squared					0.003	-0.001	0.007	0.140
Mental health	0.013	0.008	0.019	<0.001	0.008	0.002	0.015	0.009
Mental health-squared					0.005	0.001	0.009	0.008
Voted for right-wing populist party [a]								
Self-assessed health	0.024	0.014	0.033	<0.001	0.026	0.017	0.036	<0.001
Self-assessed health-squared					0.010	0.003	0.017	0.006
Mental health	0.012	0.003	0.020	0.006	0.010	0.000	0.020	0.058
Mental health-squared					0.002	-0.005	0.008	0.561
Voted for left-wing populist party [a]								
Self-assessed health	0.029	0.019	0.039	<0.001	0.032	0.022	0.042	<0.001
Self-assessed health-squared					0.011	0.004	0.019	0.003
Mental health	0.021	0.012	0.030	<0.001	0.016	0.005	0.027	0.005
Mental health-squared	0.034	0.005	0.063		0.006	-0.001	0.013	0.113

Note: A higher score on the health variables indicates poorer health.
[a] The analysis only included data corresponding to the elections in 2010, 2012 and 2017.

Table S3. Individual associations between changes in health and changes in the support for populist parties using fixed effects logistic models.

	Model 1				Model 2			
**Outcome and health variables**	**b**	**95% CI**		***p*-value**	**b**	**95% CI**		***p*-value**
Intention to vote for right-wing populist party								
Self-assessed health	-0.029	-0.213	0.155	0.759	-0.034	-0.219	0.150	0.715
Self-assessed health-squared					0.129	-0.001	0.259	0.052
Mental health	0.071	-0.079	0.220	0.355	0.011	-0.177	0.200	0.906
Mental health-squared					0.052	-0.049	0.153	0.311
Intention to vote left-wing populist party								
Self-assessed health	0.084	-0.094	0.261	0.356	0.087	-0.091	0.265	0.339
Self-assessed health-squared					-0.039	-0.160	0.083	0.534
Mental health	-0.030	-0.169	0.109	0.670	-0.087	-0.269	0.095	0.349
Mental health-squared					0.044	-0.048	0.137	0.345
Voted for right-wing populist party [a]								
Self-assessed health	0.248	-0.232	0.728	0.311	0.173	-0.320	0.666	0.491
Self-assessed health-squared					0.256	-0.113	0.626	0.174
Mental health	-0.072	-0.422	0.277	0.685	-0.006	-0.455	0.443	0.980
Mental health-squared					-0.054	-0.282	0.175	0.645
Voted for left-wing populist party [a]								
Self-assessed health	0.118	-0.242	0.477	0.521	0.113	-0.247	0.474	0.538
Self-assessed health-squared					0.042	-0.179	0.264	0.707
Mental health	0.169	-0.129	0.467	0.266	0.139	-0.229	0.507	0.459
Mental health-squared					0.028	-0.174	0.231	0.785

Note: A higher score on the health variables indicates poorer health. All the models were adjusted for year dummies and the following covariates: age, partner, number of children, log household income, work and financial difficulties.

[a] The analysis only included data corresponding to the three elections in 2010, 2012 and 2017; iterations were set at max. 100.
